# Pregnancy and future health: Why do we need to spread the word?

**DOI:** 10.1111/aogs.14551

**Published:** 2023-04-04

**Authors:** Jens Fuglsang

**Affiliations:** ^1^ Department of Obstetrics and Gynecology Aarhus University Hospital Aarhus Denmark; ^2^ Department of Clinical Medicine Aarhus University Aarhus Denmark

In this issue of AOGS, a narrative review by McNestry and colleagues demonstrates the consequences of pregnancy complications for the maternal health later in life.^1^ Much attention has been paid to the risk of future health problems for an individual whose time in utero has been subjected to suboptimal conditions. The developmental origins of health and disease hypothesis has gained much popularity.^2^ However, not only the newborn may face later consequences after a complicated course of pregnancy. In the last decades, more and more research has generated evidence of an association between gestational diabetes mellitus (GDM) and later type 2 diabetes. Also, the risk of later cardiovascular disease after pregnancies complicated by hypertensive disorders has been demonstrated. In the review by McNestry et al.^1^ associations between many more pregnancy complications, including early pregnancy complications and later health are demonstrated. This opens an opportunity to stratify risk, and start preventive measures early in those at increased risk.

What can be done, then?

Numerous approaches may be worthwhile considering. For some of the disease entities there is an increased risk of recurrence. For example, pre‐eclampsia may develop in a subsequent pregnancy and therefore, prophylaxis with acetylsalicylic acid starting in the first trimester may be recommended. Similarly, as GDM may also recur in subsequent pregnancies, the inter‐pregnancy period would be an opportunity to try to mitigate the development of GDM or to minimize the impact of GDM developing in a new pregnancy through life style changes.

For a large array of pregnancy complications, later health problems develop years after pregnancy. Efforts should then be made for either prophylaxis, if possible, or for early detection and treatment. This of course requires that prophylactic measures or/and a treatment is available.

One obvious prophylactic measure would be to recommend, to enable, to support, and to sustain breastfeeding. Breastfeeding has numerous advantages for both the newborn and the mother. For example, lactation is associated with a lower risk for the mother of later breast cancer, hypertension and cardiovascular disease.^3^, ^4^ The mechanisms behind are far from understood but this should not prevent efforts that could optimize the possibilities for engaging women in lactation and breastfeeding. Interestingly, government health authorities have had a tendency to recommend breastfeeding for a period of time that corresponds very well with the societal possibilities for mothers being on maternity leave. In the Scandinavian countries, mothers in general have very good possibilities for maternity leave. In large parts of the World, however, maternity leave is a luxury that many women will not have, at least not for a longer period of time. Supporting maternity care and mothers' rights may thus be a way to ensure better future health for both mother and baby.^5^

Another approach to counteract later adverse health effects after complicated pregnancies is to increase the awareness of pregnancy as a harbinger of later heath. Many general practitioners, internists, cardiologists, and so forth, may not be fully aware of this association. Several other specialists, such as the fertility clinic physicians, obstetricians, neonatologists, and epidemiologists have an obligation to bring forth knowledge of these associations. This knowledge should be disseminated throughout the society, from policy makers and health authorities to primary caregivers.

Sexual health includes non‐communicable diseases. Educational efforts directed towards adolescents and young adults might also be worthwhile. The awareness of pregnancy being a ‘stress test’ for later health may prove to be of tremendous importance for a woman in her reproductive age. Her future may benefit from it.
